# Supplementary material for: Statistical modeling of SARS-CoV-2 substitution processes: predicting the next variant
Source: Commun Biol. 2022 Mar 29;5:285. doi: 10.1038/s42003-022-03198-y (PMC8964801; doi:10.1038/s42003-022-03198-y)
Supplement: Supplementary file 2 — Supplementary Information [file 42003_2022_3198_MOESM2_ESM.pdf]

Statistical modeling of SARS-CoV-2 substitution processes: predicting the next variant - Supplementary Information

Keren Levinstein Hallak[1] and Saharon Rosset[1,*]

[1] *Department of Statistics and Operations Research, School of Mathematical Sciences, Tel-Aviv University, 6997801, Tel-Aviv, Israel*

[*] *Corresponding author, E-mail: saharon@tauex.tau.ac.il*

Supplementary Note 1 - NCBI phylogenetic tree

We perform the same analysis on the phylogenetic tree reconstructed by NCBI[1,2] as was done in the main paper for the tree we reconstructed by applying the sarscov2phylo method by Lanfear[3] (see main paper, Figure 1, Figure 3 and Figure 4). The NCBI dataset contains 38,277 sequences that passed quality control out of the 61,835 sequences that were available online[4] on February 8th, 2021. The results are given in Figure S1, Figure S3 and Figure S5. The three highest-ranking models for the NCBI tree are different from those obtained in the main paper. However, the ranks of the NCBI highest-ranking models according to the phylogenetic tree reconstructed by the sarscov2phylo method are relatively high (585, 395, and 51 out of 43,254) and also vice versa, the ranks of the highest-ranking models in the main paper are relatively high according to NCBI's models ranking (1317, 88 and 2853 out of 43,254).

The results in Figure S3 and Figure S5 highly resemble these from the main paper and follow the same analysis therein, confirming the robustness of our method.

Supplementary Note 2 - Additional test set

In order to further validate our model over a time period that is not adjacent to the training time period, we evaluated the ability of our top models to predict novel substitutions in the time period of 9/15/2021-10/1/2021. We followed the same procedure described in the predictions section in the paper: We considered the 10,557 *test* sequences that were added to the NCBI database in this period and were not excluded by the alignment procedure (as described in the Methods section). We then identified 10,144 sites that had at most one base different from the base appearing in the training data, allowing us to confidently identify the substitution that occurred without inferring a phylogenetic tree for the test sequences. In these, we identified 1,258 sites that had at least one substitution in the test sequences. To avoid labeling sequencing errors, we required a minimum of two different test sequences with the mutated state; hence only 567 sites remained. Sites that had a single test sample with a mutated state were entirely ignored in the evaluation phase. Figure S6 provides the results for our top ten models, while Figure S7 provides the lift curves derived from the third Poisson model of non-synonymous amino acid substitutions. This confirms the robustness of our method as we obtain results very similar to those mentioned in the main text.

Supplementary Note 3 - Ancestral sequence reconstruction method validation

We examined our ancestral sequence reconstruction method using the following tests: First, we examined the number of adjacent back substitutions for each tree. We define *adjacent back substitutions* as substitutions from one base to another base and then back to the original base at the following node. In the tree reconstructed according to Lanfear's method, there were no adjacent back substitutions, while in the tree reconstructed by NCBI, there were only two with no obvious alternative (examined manually). Next, we counted the number of *back substitutions* (defined as substitutions back to the ancestral state as appears in the reference sequence). According to Lanfear's method, the total number of back substitutions in the reconstructed tree is 991, out of 51,527 substitutions (1.92%), and in the tree reconstructed by NCBI, there are 876 back substitutions out of 47,809 sub-

stitutions (1.83%). Our ancestral sequence reconstruction method handles transitions and transversions identically. Hence, observing a ti/tv ratio in the back substitutions similar to the general observed ti/tv ratio indicates that our method performs well. For all sites that had only two possible bases along the tree, we counted the number of back transitions, back transversions, and the total number of transitions and transversions. For the tree reconstructed by Lanfear, the ti/tv ratio is 3.07 for all substitutions and 3.36 for back substitutions. For the NCBI tree, the ti/tv ratio is 3.45 for all substitutions and 3.49 for back substitutions. A chi-squared test supports the null hypothesis according to which the number of back substitutions out of the total number of substitutions does not depend on the classification to transitions/transversions with p-values of 0.2453 and 0.9247 for the Lanfear and NCBI trees correspondingly.

Supplementary References

1. National Center for Biotechnology Information. `https://www.ncbi.nlm.nih.gov/labs/virus/vssi/#/precomptree`. 2021.
2. Benson, D. *et al.* GenBank Nucleic Acids Res 41 (D1). *D36–D42* (2013).
3. Lanfear, R. `https://github.com/roblanf/sarscov2phylo`. 2021.
4. National Center for Biotechnology Information. `https://www.ncbi.nlm.nih.gov/sars-cov-2/`. 2021.

Figure S1: **Top-scoring models for the training dataset for NCBI's reconstructed phylogenetic tree.**

- \- Omission
- \+ Inclusion
- / Division

	Gene	Nucleotide	Amino Acid	Codon	Codon position	Mature Peptide	Stem Loop	CG Pair	Right Neighbor	Left Neighbor	# of Sub-Models
First models ranked by AIC	-	/	/	-	+	/	-	-	+	-	219
	-	/	-	/	-	+	-	-	+	+	200
	-	/	/	-	/	/	-	-	+	+	275
First models ranked by Poisson AIC	+	-	-	/	+	/	-	-	+	+	248
	+	-	-	/	+	/	+	-	+	+	248
	+	-	-	/	+	/	/	-	+	+	341

The first three rows correspond to the top-scoring models when NB regression is applied. The next three rows correspond to the top-scoring models when Poisson regression is used. Each explaining factor is either (−) omitted from the model, (+) used as an explanatory factor, or (/) used to split the GLM into sub-models.

Figure S2: **Top ten scoring models for the training dataset.**

Legend: - Omission; + Inclusion; / Division

	Gene	Nucleotide	Amino Acid	Codon	Codon Position	Mature Peptide	Stem Loop	CG Pair	Right Neighbor	Left Neighbor	# of Sub-Models
First models ranked by NB AIC	-	/	/	-	/	/	+	/	-	-	356
	-	/	/	-	/	/	+	/	+	+	356
	+	/	/	-	/	/	+	/	-	-	356
	-	/	/	-	/	/	-	/	-	-	356
	-	/	/	-	/	/	-	/	+	+	356
	+	/	/	-	/	/	+	/	+	+	356
	-	+	/	-	/	/	-	/	+	+	240
	+	+	/	-	/	/	-	/	+	+	240
	+	/	/	-	/	/	-	/	-	-	356
	-	+	/	-	/	/	-	/	-	-	240
First models ranked by Poisson AIC	+	-	-	/	+	/	+	/	+	+	370
	+	/	/	-	/	/	+	/	+	+	356
	+	-	-	/	/	-	+	-	/	/	724
	+	-	-	/	/	-	+	+	/	/	724
	+	-	-	/	/	-	+	/	/	/	746
	+	/	/	-	/	/	-	/	+	+	356
	+	+	-	/	-	/	+	/	+	+	370
	+	-	-	/	/	-	+	-	+	+	231
	+	/	-	/	-	-	+	-	+	+	200
	+	-	-	/	+	/	-	/	+	+	370

The first ten rows correspond to the top-scoring models when NB regression is applied. The next ten rows correspond to the top-scoring models when Poisson regression is used. Each explaining factor is either (−) omitted from the model, (+) used as an explanatory factor, or (/) used to split the GLM into sub-models.

Figure S3: **Prediction results for the top three models for NCBI's reconstructed phylogenetic tree.**

	Model #	Non-synonymous amino acid substitutions						Synonymous amino acid substitutions					
		Poisson			Negative Binomial			Poisson			Negative Binomial		
		AUC	3% Lift Vs. Random model	3% Lift Vs. Base model	AUC	3% Lift Vs. Random model	3% Lift Vs. Base model	AUC	3% Lift Vs. Random model	3% Lift Vs. Base model	AUC	3% Lift Vs. Random model	3% Lift Vs. Base model
All genes	1	0.833	5.408	2.298	0.815	3.205	1.362	0.861	3.747	1.500	0.859	3.236	1.326
	2	0.832	5.308	2.255	0.815	3.004	1.277	0.865	3.804	1.523	0.863	3.634	1.488
	3	0.834	4.506	1.915	0.808	2.053	0.872	0.861	3.747	1.500	0.859	3.293	1.349
Spike gene	1	0.825	4.062	2.667	0.788	3.046	2.000	0.860	3.798	2.667	0.854	2.374	1.667
	2	0.821	4.569	3.000	0.786	3.046	2.000	0.880	4.273	3.000	0.874	3.798	2.667
	3	0.814	4.062	2.667	0.759	1.015	0.667	0.859	3.798	2.667	0.853	2.849	2.000

We use the top three Poisson and Negative Binomial models from Figure S1 for prediction on the test dataset. Results for the entire genome are in the first three rows, for the spike protein only in the last three. Results are shown separately for predicting non-synonymous amino acid substitutions (left half) and predicting synonymous substitutions (right half, these results are not discussed in the text). The first column in each quarter of the table shows the area under the ROC curve (AUC) for the corresponding prediction task and modeling approach. We highlighted the top-scoring model for every (substitution type, locus, approach) combination. Overall we obtained high AUC scores, showing the models successfully predicted many of the substitutions. The second and third columns in each quarter are 3% lift scores of each model versus the random model and the more elaborate base model (see text and Methods). The top models significantly outperform both baselines stressing the benefits of our approach over more naive statistical predictions. The model presented in Figure S5 (third Poisson model for non-synonymous amino acid substitutions) is also red-framed.

Figure S4: **Prediction results for the top ten models.**

	Model #	Non-synonymous amino acid substitutions						Synonymous amino acid substitutions					
		Poisson			Negative Binomial			Poisson			Negative Binomial		
		AUC	3% Lift Vs.		AUC	3% Lift Vs.		AUC	3% Lift Vs.		AUC	3% Lift Vs.	
			Random model	Base model		Random model	Base model		Random model	Base model		Random model	Base model
All genes	1	0.835	4.707	2.238	0.821	4.607	1.957	0.858	3.577	1.465	0.856	3.577	1.432
	2	0.832	4.406	2.095	0.819	4.306	1.830	0.861	3.861	1.581	0.858	3.463	1.386
	3	0.836	5.358	2.548	0.826	4.557	1.936	0.847	3.520	1.442	0.846	3.690	1.477
	4	0.835	4.757	2.262	0.821	4.657	1.979	0.859	3.634	1.488	0.857	3.577	1.432
	5	0.833	4.406	2.095	0.819	4.356	1.851	0.862	3.747	1.535	0.859	3.463	1.386
	6	0.833	4.957	2.357	0.825	4.557	1.936	0.849	3.520	1.442	0.848	3.463	1.386
	7	0.833	4.406	2.095	0.820	4.456	1.894	0.855	3.577	1.465	0.854	3.577	1.432
	8	0.834	4.907	2.333	0.826	4.557	1.936	0.849	3.634	1.488	0.850	3.634	1.455
	9	0.836	5.308	2.524	0.826	4.506	1.915	0.849	3.463	1.419	0.848	3.690	1.477
	10	0.835	4.757	2.262	0.822	4.657	1.979	0.859	3.634	1.488	0.858	3.577	1.432
Spike gene	1	0.814	4.062	2.667	0.786	2.538	1.250	0.867	4.748	3.333	0.861	1.899	1.333
	2	0.814	4.062	2.667	0.781	3.554	1.750	0.864	4.273	3.000	0.859	3.798	2.667
	3	0.830	4.062	2.667	0.827	3.554	1.750	0.863	4.748	3.333	0.864	4.748	3.333
	4	0.813	4.062	2.667	0.785	2.538	1.250	0.868	4.748	3.333	0.863	1.899	1.333
	5	0.813	4.062	2.667	0.780	3.554	1.750	0.864	3.798	2.667	0.859	3.323	2.333
	6	0.826	4.062	2.667	0.823	3.046	1.500	0.849	3.323	2.333	0.848	3.323	2.333
	7	0.811	4.062	2.667	0.779	3.554	1.750	0.847	3.798	2.667	0.849	3.323	2.333
	8	0.823	4.062	2.667	0.820	3.046	1.500	0.855	4.273	3.000	0.849	4.273	3.000
	9	0.830	4.062	2.667	0.827	3.554	1.750	0.863	4.748	3.333	0.863	4.748	3.333
	10	0.814	4.062	2.667	0.785	3.046	1.500	0.868	4.748	3.333	0.866	3.798	2.667

We use the top ten Poisson and Negative Binomial models from Figure S2 for prediction on the test dataset. Results for the entire genome are in the first ten rows, for the spike protein only in the last ten. Results are shown separately for predicting non-synonymous amino acid substitutions (left half) and predicting synonymous substitutions (right half, these results are not discussed in the text). The first column in each quarter of the table shows the area under the ROC curve (AUC) for the corresponding prediction task and modeling approach. We highlighted the top-scoring model for every (substitution type, locus, approach) combination. Overall we obtained high AUC scores, showing the models successfully predicted many of the substitutions. The second and third columns in each quarter are 3% lift scores of each model versus the random model and the more elaborate base model (see text and Methods). The top models significantly outperform both baselines stressing the benefits of our approach over more naive statistical predictions. The model we analyzed further in the text (third Poisson model for non-synonymous amino acid substitutions) is also red-framed.

Figure S5: **Lift curves of the winning model versus the random (red) and base models (cyan) for NCBI's reconstructed phylogenetic tree.**

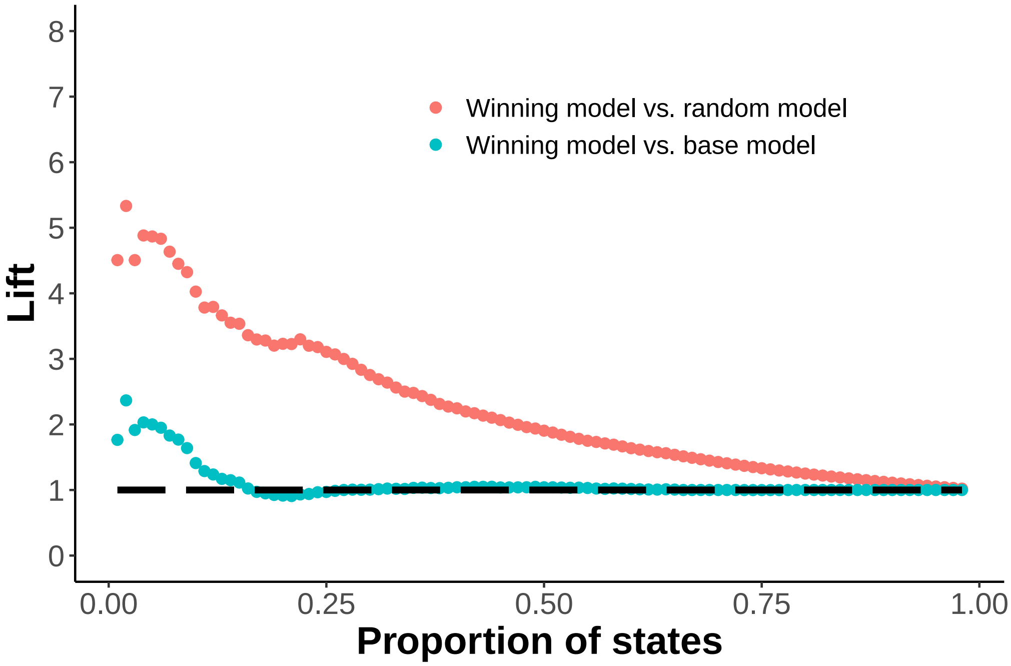

We compare the winning model, the third-ranked Poisson model of non-synonymous amino acid substitutions, against two baselines. The first is the random ordering of all possible relevant substitutions (red), and the second is a base model, which considers the exposure and the transition/transversion (ti/tv) ratio, but not the other explanatory factors (cyan). To compare, we show the lift score (the ratio of true positives compared to a baseline model) as a function of the proportion of states considered. Source data: Supplementary Data 4.

Figure S6: **Prediction results for the top ten models for the additional test set of sequences collected between September 15th, 2021, and October 1st, 2021**

	Model #	Non-synonymous amino acid substitutions						Synonymous amino acid substitutions					
		Poisson			Negative Binomial			Poisson			Negative Binomial		
		AUC	3% Lift Vs. Random model	3% Lift Vs. Base model	AUC	3% Lift Vs. Random model	3% Lift Vs. Base model	AUC	3% Lift Vs. Random model	3% Lift Vs. Base model	AUC	3% Lift Vs. Random model	3% Lift Vs. Base model
All genes	1	0.836	5.578	3.118	0.821	5.262	2.941	0.828	4.297	1.778	0.824	3.491	1.368
	2	0.838	5.367	3.000	0.827	5.051	2.824	0.825	4.431	1.833	0.821	4.028	1.579
	3	0.833	5.999	3.353	0.824	5.578	3.118	0.810	3.491	1.444	0.808	3.626	1.421
	4	0.836	5.578	3.118	0.820	5.157	2.882	0.827	4.297	1.778	0.823	3.357	1.316
	5	0.837	5.367	3.000	0.826	5.051	2.824	0.824	4.566	1.889	0.820	3.626	1.421
	6	0.835	5.578	3.118	0.829	5.578	3.118	0.804	4.163	1.722	0.802	3.894	1.526
	7	0.837	5.367	3.000	0.826	5.157	2.882	0.819	4.163	1.722	0.818	3.491	1.368
	8	0.840	5.578	3.118	0.833	5.578	3.118	0.809	3.626	1.500	0.810	3.626	1.421
	9	0.833	5.999	3.353	0.822	5.472	3.059	0.810	3.491	1.444	0.808	3.626	1.421
	10	0.836	5.578	3.118	0.821	5.157	2.882	0.827	4.297	1.778	0.824	3.357	1.316
Spike gene	1	0.766	4.184	5.000	0.735	3.347	2.000	0.831	3.985	3.000	0.812	0.000	0.000
	2	0.775	4.184	5.000	0.746	4.184	2.500	0.808	5.314	4.000	0.794	3.985	3.000
	3	0.786	4.184	5.000	0.781	3.347	2.000	0.851	3.985	3.000	0.847	3.985	3.000
	4	0.768	4.184	5.000	0.734	3.347	2.000	0.831	3.985	3.000	0.812	0.000	0.000
	5	0.775	4.184	5.000	0.745	4.184	2.500	0.807	5.314	4.000	0.792	3.985	3.000
	6	0.798	4.184	5.000	0.796	3.347	2.000	0.814	3.985	3.000	0.800	3.985	3.000
	7	0.772	4.184	5.000	0.742	3.347	2.000	0.808	5.314	4.000	0.803	3.985	3.000
	8	0.793	4.184	5.000	0.791	3.347	2.000	0.792	5.314	4.000	0.798	3.985	3.000
	9	0.786	4.184	5.000	0.781	3.347	2.000	0.851	3.985	3.000	0.847	3.985	3.000
	10	0.768	4.184	5.000	0.734	3.347	2.000	0.831	3.985	3.000	0.817	3.985	3.000

We use the top ten Poisson and Negative Binomial models from Figure S2 for prediction on the additional test dataset. Results for the entire genome are in the first ten rows, for the spike protein only in the last ten. Results are shown separately for predicting non-synonymous amino acid substitutions (left half) and predicting synonymous substitutions (right half, these results are not discussed in the text). The first column in each quarter of the table shows the area under the ROC curve (AUC) for the corresponding prediction task and modeling approach. We highlighted the top-scoring model for every (substitution type, locus, approach) combination. Overall we obtained high AUC scores, showing the models successfully predicted many of the substitutions. The second and third columns in each quarter are 3% lift scores of each model versus the random model and the more elaborate base model (see text and Methods). The top models significantly outperform both baselines stressing the benefits of our approach over more naive statistical predictions. The model presented in Figure S7 (third Poisson model for non-synonymous amino acid substitutions) is also red-framed.

Figure S7: **Lift curves of the winning model versus the random (red) and base models (cyan) for the additional test set of sequences collected between September 15th, 2021, and October 1st, 2021.**

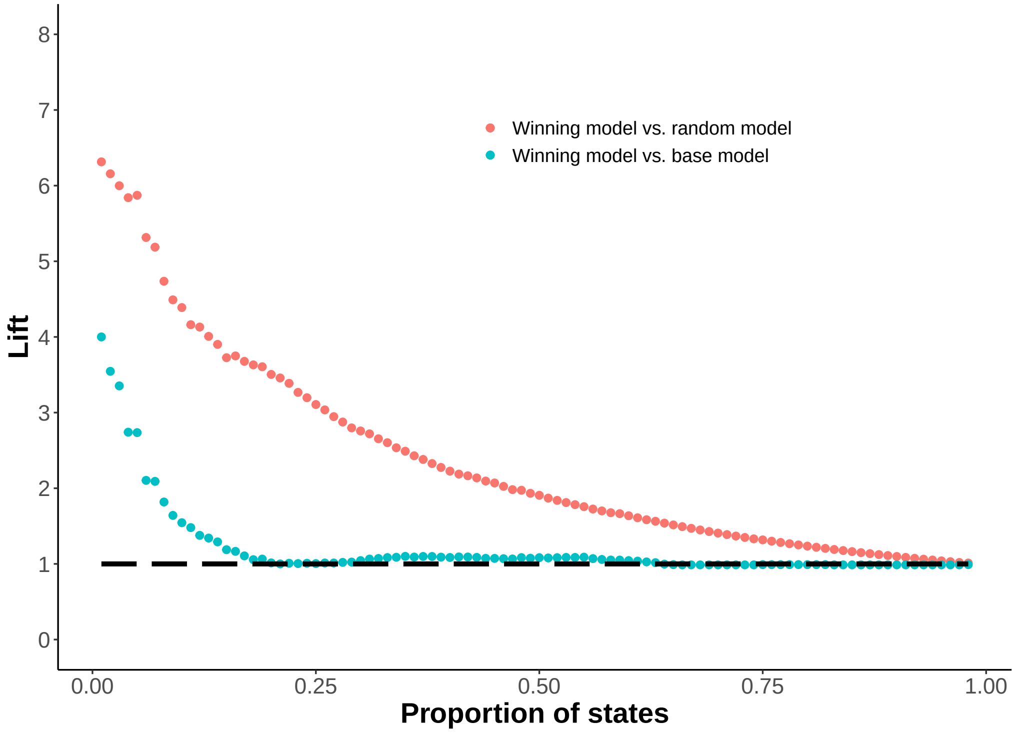

We compare the winning model, the third-ranked Poisson model of non-synonymous amino acid substitutions, against two baselines. The first is the random ordering of all possible relevant substitutions (red), and the second is a base model, which considers the exposure and the transition/transversion (ti/tv) ratio, but not the other explanatory factors (cyan). To compare, we show the lift score (the ratio of true positives compared to a baseline model) as a function of the proportion of states considered. Source data: Supplementary Data 5.

Figure S8: **Rank of spike protein amino acid substitutions.**

Substitution	Rank
677H	15
1101Y	41
655Y	42
1027I	129
76I	145
95I	163
716I	167
417N	195
950N	300
375F	406
701V	590
67V	604
477N	613
981F	691
18F	713
26S	769
1092K	797
154K	803
484K	821
339D	871
142D	915
346K	930
190S	1168
215G	1228

Substitution	Rank
253G	1282
496S	1283
446S	1290
547K	1439
19R	1456
859N	1470
20N	1471
478K	1479
138Y	1586
52R	1755
493R	1767
498R	1768
796Y	1856
1118H	1892
13I	1950
888L	1973
969K	2020
440K	2040
679K	2041
1176F	2400
570D	2572
681R	2698
681H	2701
954H	2733

Substitution	Rank
1071H	2735
505H	3122
373P	3186
764K	3274
484Q	3402
75V	3735
246I	3829
158G	4443
490S	4667
80A	4793
484A	5328
417T	5419
152C	5535
145N	5950
982A	6106
212I	6406
614G	6424
452Q	6597
501Y	6600
144S	7185
452R	7390
856K	8023
371L	9590
5F	-

Ranking was performed by our prediction model on 13,544 possible non-synonymous amino acid substitutions in the spike protein resulting from one nucleotide change. Shown are the ranks of the 72 substitutions comprising the following variants: Alpha (lineage B.1.1.7), Beta (lineage B.1.351), Gamma (lineage P.1), Delta (lineage B.1.617.2), Theta (lineage P.3), Omicron (lineage B.1.1.529), Lambda (lineage C.37), Mu (lineage B.1.621), Epsilon (lineages B.1.429, B.1.427), Zeta (lineage P.2), Eta (lineage B.1.525) and Theta (lineage P.3). The highlighted substitutions were not part of the training dataset. The substitution 5F does not appear in our list of 13,544 possible substitutions since it requires a substitution in more than one nucleotide for the sequences in our training dataset.
