# Supplementary material for: Statistical modeling of SARS-CoV-2 substitution processes: predicting the next variant
Source: Commun Biol. 2022 Mar 29;5:285. doi: 10.1038/s42003-022-03198-y (PMC8964801; doi:10.1038/s42003-022-03198-y)
Supplement: Supplementary file 3 — Description of Additional Supplementary Files [file 42003_2022_3198_MOESM3_ESM.pdf]

Description of Additional Supplementary Files

File name: Supplementary Data 1
Description: All models fitted to the training data obtained from the phylogenetic tree we reconstructed according to Lanfear's method. The models are ranked according to the minimum of their Negative Binomial (NB) and Poisson AIC scores. Each explaining factor is either (–) omitted from the model, (+) used as an explanatory factor, or (/) used to split the GLM into submodels.

File name: Supplementary Data 2
Description: All models fitted to the training data obtained from the phylogenetic tree reconstructed by NCBI. The models are ranked according to the minimum of their Negative Binomial (NB) and Poisson AIC scores. Each explaining factor is either (–) omitted from the model, (+) used as an explanatory factor, or (/) used to split the GLM into submodels.

File name: Supplementary Data 3
Description: Predicted rates for non-synonymous amino acid substitutions in the spike protein. The last two columns indicate whether the amino acid-location pair was Page 7 of 15 observed in the training and test sequences.

File name: Supplementary Data 4
Description: Source data for Figure 4.

File name: Supplementary Data 5
Description: Source data for Supplementary Figure S3.

File name: Supplementary Data 6
Description: Source data for Supplementary Figure S7.
